# Hemodynamic behavior in a randomized trial of intensive alveolar recruitment after cardiac surgery

**DOI:** 10.1186/cc14274

**Published:** 2015-03-16

**Authors:** A Leme, M Amato, E Osawa, J Fukushima, M Feltrim, E Nozawa, J Almeida, L Hajjar, F Galas

**Affiliations:** 1 Heart Institute, São Paulo, Brazil

## Introduction

The potential benefits of a protocol of intensive alveolar recruitment may be outweighed by its detrimental effects in hemodynamic stability after cardiac surgery. The aim of this study was to analyze the hemodynamic behavior of patients included in a trial of intensive alveolar recruitment after cardiac surgery.

## Methods

In this randomized trial, we assigned adult patients with PaO_2_/ FIO_2_ <250 at a PEEP of 5 cmH_2_O to either intensive alveolar recruitment or a standard protocol, both using low-tidal volume ventilation (6 ml/ kg/ibw) after adequate volemia status. Our hypothesis was that an intensive alveolar recruitment protocol with controlled pressure of 15 cmH_2_O and PEEP of 30 cmH_2_O during 1 minute, repeated three times at 1-minute intervals between each maneuver, would not cause hemodynamic instability.

## Results

**Figure 1:**
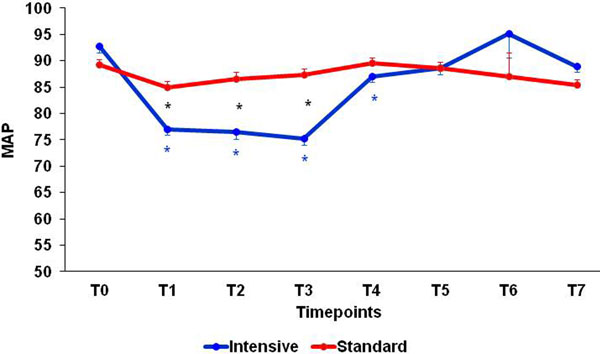


In total, 163 patients were included in the standard and 157 in the intensive group. Patients of the intensive group had a significant reduction of the MAP at T1, T2 and T3 (1 hour, 2 hours and 3 hours of the protocol), returning to baseline after T4 (Figure 1). No patients had severe hypotension (MAP <65 mmHg) and the study was not stopped in any case. The length of the hospital stay was shorter among patients in the intensive group (10.9 (9.9 to 11.9) vs. 12.4 days (11.3 to 13.6); *P *= 0.045).

## Conclusion

An intensive alveolar recruitment protocol did not result in hemodynamic instability in hypoxemic patients after cardiac surgery (NCT01502332).

